# Dilemma after termination of pregnancy due to urogenital fetal anomalies: Discrepancy between prenatal ultrasonographic diagnosis and autopsy

**DOI:** 10.1002/ijgo.14083

**Published:** 2022-01-15

**Authors:** Ozge Ozdemir, Figen Aksoy, Cihat Sen

**Affiliations:** ^1^ Department of Obstetrics and Gynecology Division of Maternal‐Fetal Medicine Istanbul University‐Cerrahpasa Cerrahpasa Medical Faculty Istanbul Turkey; ^2^ Department of Pathology Istanbul University‐Cerrahpasa Cerrahpasa Medical Faculty Istanbul Turkey

**Keywords:** discrepancy, fetal autopsy, fetal urogenital anomaly, pregnancy termination, prenatal ultrasound, value of autopsy

## Abstract

**Objective:**

To evaluate the agreement and disagreement between prenatal ultrasound and fetal autopsy findings in pregnancy terminations due to urogenital anomalies.

**Methods:**

Of 453 pregnancy terminations performed due to fetal anomalies, 82 cases with urogenital anomalies on either prenatal ultrasound or fetal autopsy were included in this retrospective study. The discrepancy between prenatal ultrasound and fetal autopsy findings on urogenital anomaly findings was evaluated.

**Results:**

Complete agreement between prenatal ultrasound and fetal autopsy findings was noted in 33 (40.2%) cases (particularly for megacystis, bilateral renal agenesis, and infantile polycystic kidney), whereas partial agreement (anal atresia and horseshoe kidney as additional minor findings) and altered diagnosis were noted in 12 (14.6%) and 8 (9.8%) cases, respectively. Disagreement was noted in 29 (35.4%) cases including anomaly only on autopsy in 20 (24.3%) cases (renal agenesis, horseshoe kidney and multicystic dysplastic kidney in particular) and anomaly only on ultrasound in 9 (10.9%) cases.

**Conclusions:**

Accordingly, our findings indicate fetal autopsy to be a method of vital importance in complementing prenatal diagnosis; it may add valuable information that may improve future pregnancy management and counseling of parents, and hence prenatal ultrasound and fetal autopsy should be regarded as complementary techniques.

## 1 INTRODUCTION

Technical advancements in imaging techniques have enabled high‐resolution prenatal ultrasonography with a higher diagnostic accuracy in detection rate of fetal malformations.^1^, ^2^, ^3^, ^4^ Nonetheless, in pregnancy terminations based on prenatal prediction of fetal anomalies, fetal autopsy is important in terms of assessing the quality and accuracy of prenatal ultrasound findings on fetal anomaly as well as for the potential implications of detected malformations for future pregnancies.^1^, ^2^, ^3^, ^4^ Hence, a prenatal ultrasound examination and a subsequent detailed fetal autopsy provide the basis for a correct diagnosis in pregnancies terminated because of congenital anomalies; fetal autopsy is regarded as the reference standard for assessing the accuracy of prenatal ultrasound.^3^, ^5^, ^6^, ^7^, ^8^

Given that both methods have intrinsic limitations—with not all autopsy findings being detectable by ultrasound and vice versa—studies comparing ultrasound and autopsy findings are considered important to reveal the level of accuracy of the prenatal diagnosis. This is valuable not only for parents and physicians but also for genetic counseling, determination of recurrence risk, and epidemiologic analysis.^1^, ^2^, ^4^, ^5^

Although congenital urinary system anomalies represent a large group of fetal anomalies, most studies have addressed the prenatal detection rates of overall major urinary system anomalies rather than specifically focusing on particular organ systems or minor renal anomalies.^1^, ^2^, ^3^, ^4^, ^5^, ^9^

This study aimed to investigate the agreement and disagreement between prenatal ultrasound and fetal autopsy findings in pregnancy terminations specifically due to urogenital anomalies.

## 2 MATERIALS AND METHODS

### 2.1 Study population

Of 453 pregnancy terminations performed for fetal anomalies between January 2001 and January 2017 in our tertiary care clinic, 82 cases with urogenital anomalies on prenatal ultrasound (*n* = 62) or only on fetal autopsy (*n* = 20) were included in this retrospective study. The agreement between prenatal ultrasound and fetal autopsy findings on urogenital anomaly findings was evaluated.

Written informed consent was obtained from the parent/legal guardian of each patient following a detailed explanation of the objectives and protocol of the study which was conducted in accordance with the ethical principles stated in the Declaration of Helsinki and approved by the Istanbul University‐Cerrahpasa, Cerrahpasa Medical Faculty Clinical Research Ethics Committee (date of approval June 15, 2017, no. 227794.A‐49).

### 2.2 Assessments

The correlations between the prenatal ultrasound and fetal autopsy findings were classified into five levels of agreement: complete agreement, partial agreement (addition of minor findings on autopsy), and altered diagnosis after autopsy, disagreement with positive findings only on autopsy, and disagreement with positive findings only on ultrasound.

### 2.3 Prenatal ultrasound and fetal autopsy examination

All prenatal ultrasound examinations were performed using the Toshiba Xario Diagnostic Ultrasound System (Toshiba Medical Systems Corp.) between January 2001 and Aug 2016 and using the Voluson E10 USG machine (GE Healthcare Systems, WI, USA) after August 2016. Termination of pregnancy was performed in cases with major anomalies before fetal viability (<24 weeks of pregnancy). All post‐mortem examinations were carried out in the pathology department by a perinatal pathologist. A full and standard fetal autopsy including photography, whole‐body X‐ray scan, gross examination, dissection, and histologic examination of fetal organs was performed after obtaining informed consent from the parents.

### 2.4 Statistical analysis

Descriptive statistics were reported including frequencies and percentages for categorical variables.

## 3 RESULTS

### 3.1 Prenatal ultrasound findings

Megacystis (*n* = 14), bilateral renal agenesis (*n* = 9), and infantile polycystic kidney (*n* = 8) were the most common urogenital anomalies detected on prenatal ultrasound. Central nervous system anomalies were the concomitant anomaly in 16 cases.

### 3.2 Level of agreement between prenatal ultrasonography and fetal autopsy

Complete agreement was evident between prenatal ultrasound and fetal autopsy findings in 33 (40.2%) cases, whereas partial agreement and altered diagnosis were noted in 12 (14.6%) and 8 (9.8%) cases, respectively.

Disagreement was noted in 29 (35.4%) cases including anomaly only on autopsy in 20 (24.3%) cases and anomaly only on ultrasound in 9 (10.9%) cases.

### 3.3 Specific diagnoses according to level of agreement

Megacystis (*n* = 7), infantile polycystic kidney (*n* = 5), and bilateral renal agenesis (*n* = 5) were the three most common diagnoses confirmed by both prenatal ultrasound and fetal autopsy (Table 1).

**TABLE 1:** Specific diagnoses according to level of agreement between prenatal ultrasonography and fetal autopsy

Complete agreement (same diagnosis on ultrasound and autopsy) (*n* = 33)	
Megacystis	*n* = 7
Infantile polycystic kidney	*n* = 5
Bilateral renal agenesis	*n* = 5
Bilateral multicystic dysplastic kidney	*n* = 3
Bilateral pelviectasis	*n* = 2
Bladder exstrophy	*n* = 3
Cloacal exstrophy	*n* = 2
Renal agenesis + unilateral multicystic dysplastic kidney	*n* = 2
Pelviectasis + megacystis	*n* = 2
Unilateral renal agenesis	*n* = 1
Horseshoe kidney	*n* = 1

Partial agreement (*n* = 12)	Additional 30 minor findings on autopsy^a^
Megacystis (*n* = 5)	Anal atresia (*n* = 8)
Bilateral renal agenesis (*n* = 2)	Horseshoe kidney (*n* = 4)
Bilateral pelviectasis (*n* = 1)	Lack of uterus (*n* = 3)
Infantile polycystic kidney (*n* = 1)	Lymphangiectasis (*n* = 1)
Bladder exstrophy (*n* = 1)	Pes equinovarus (*n* = 2)
Cloacal exstrophy (*n* = 1)	Single umbilical artery (*n* = 2)
Renal agenesis + unilateral multicystic dysplastic kidney (*n* = 1)	CCAM (*n* = 2)
	Cleft lip (*n* = 2)
	Sirenomelia (*n* = 2)
	Lack of radius (*n* = 1)
	Ear anomaly (*n* = 2)
	Oligodactyly (*n* = 1)

Ultrasonography	Autopsy
Altered diagnosis (*n* = 8)	
Cloacal exstrophy	Bladder exstrophy
Bladder exstrophy + spina bifida	OEIS syndrome
Infantile polycystic kidney	Unilateral renal agenesis + unilateral multicystic kidney
Renal agenesis	Horseshoe kidney
Renal cyst	Hydroureter
Multicystic kidney	Polycystic kidney
Bladder exstrophy	Renal agenesis + omphalocele
Cloacal exstrophy + spina bifida	OICS syndrome

Disagreement (anomaly only on autopsy) (*n* = 20)	
Renal agenesis	*n* = 5
Horseshoe kidney	*n* = 4
Multicystic dysplastic kidney	*n* = 4
Unilateral renal agenesis	*n* = 3
Infantile polycystic kidney	*n* = 2
Left kidney congenital hydronephrosis	*n* = 1
Renal agenesis + unilateral multicystic dysplastic kidney	*n* = 1

Disagreement (anomaly only on ultrasound) (*n* = 9)	
Horseshoe kidney	*n* = 3
Megacystis	*n* = 2
Infantile polycystic kidney	*n* = 1
Renal agenesis	*n* = 1
Pelviectasis + megacystis	*n* = 1
Pelviectasis	*n* = 1

Abbreviations: CCAM, congenital cystic adenoid malformation; OEIS syndrome, omphalocele, exstrophy (bladder), imperforate anus, spinal defects; OICS syndrome, omphalocele, imperforate anus, cloacal exstrophy, spinal defects; ultrasound, ultrasonography.

^a^ Overall 30 minor findings were added on autopsy in 12 cases.

For the 12 cases in the partial agreement category, anal atresia (*n* = 8) and horseshoe kidney (*n* = 4) were the most common among the 30 minor diagnoses added after the autopsy (Table 1).

For the disagreement category, renal agenesis (*n* = 5), horseshoe kidney (*n* = 4), and multicystic dysplastic kidney (*n* = 4) were the most common among the 20 anomalies detected only on autopsy, whereas horseshoe kidney (*n* = 3) and megacystis (*n* = 2) were the most common anomalies among the nine anomalies detected only on ultrasound (Table 1).

### 3.4 Details of cloacal anomaly and bladder exstrophy cases

For five cloacal anomalies detected on prenatal ultrasound, complete agreement (*n* = 2) and partial agreement (*n* = 1) were evident in three cases, but the autopsy revealed altered diagnosis in two cases, one with bladder exstrophy and the other with OICS syndrome (omphalocele, imperforate anus, cloacal exstrophy, spinal defects) (Table 2).

**TABLE 2:** Details of cloacal exstrophy and bladder exstrophy cases

Case	Prenatal ultrasound	Fetal autopsy	Level of agreement
Cloacal exstrophy (*n* = 5)			
1	Cloacal exstrophy	Bladder exstrophy	Altered diagnosis
2	Cloacal exstrophy + spina bifida	+ Omphalocele and imperforate anus (OICS syndrome)	Altered diagnosis
3	Cloacal exstrophy—Omphalocele and imperforate anus (OICS syndrome)	+ Pes equinovarus	Partial agreement (plus minor findings on autopsy)
4	Cloacal exstrophy	Cloacal exstrophy	Complete agreement
5	Cloacal exstrophy	Cloacal exstrophy	Complete agreement
Bladder exstrophy (*n* = 6)			
1	Bladder exstrophy	Bladder exstrophy	Complete agreement
2	Bladder exstrophy	Bladder exstrophy	Complete agreement
3	Bladder exstrophy	Bladder exstrophy	Complete agreement
4	Bladder exstrophy	+ Omphalocele	Partial agreement (plus minor findings on autopsy)
5	Bladder exstrophy + spina bifida	+ Omphalocele and imperforate anus (OEIS syndrome)	Altered diagnosis and recurrence risk
6	Bladder exstrophy	Renal agenesis + omphalocele	Altered diagnosis and recurrence risk

For six bladder exstrophy cases detected on prenatal ultrasound, complete agreement (*n* = 3) and partial agreement (*n* = 1) were evident in four cases, and the autopsy revealed altered diagnosis in two cases, one with OEIS syndrome (omphalocele, exstrophy [bladder], imperforate anus, spinal defects) and the other with renal agenesis + omphalocele (Table 2).

## 4 DISCUSSION

Our findings revealed that complete to partial agreement between prenatal ultrasound and fetal autopsy findings was noted in 54.8% of cases, but altered diagnosis was noted in 9.8% of cases. Disagreement between prenatal ultrasound and fetal autopsy findings was noted in 35.4% cases including anomalies detectable only on autopsy (24.3%) or only on ultrasound (10.9%).

In a study assessing 308 second‐trimester fetuses with congenital anomaly, it was reported that urinary anomaly was found in 62 (20.1%) cases along with full agreement between ultrasound and fetal autopsy findings on urinary anomalies in 45 (72.6%) cases.^2^ They also noted partial agreement between ultrasound and autopsy in 6 (9.7%) cases, whereas autopsy revealed major urinary anomalies not determined by ultrasound in 10 (16.1%) cases, and an altered diagnosis from bilateral renal agenesis on ultrasound to a horseshoe kidney on autopsy was noted in 1 (1.6%) case.^2^

In a study assessing 1029 pregnancy terminations due to fetal anomalies over a 30‐year period (from 1985 to 2014), it is reported that for the urinary system anomalies per se, there was a complete agreement between ultrasound and autopsy in 118 (74.1%) cases and partial agreement in 15 (11.1%) cases, and the discrepant findings for the urinary system were multicystic dysplastic kidneys.^5^ In another study on 408 fetuses with congenital anomalies, urinary system anomalies were reported in 112 (27%) cases along with full agreement between the prenatal ultrasound and autopsy in 97 (87%) of the 112 cases, partial agreement in 5 (4.5%) cases, and disagreement in 10 (8.9%) cases including 8 (7.2%) cases with autopsy findings not detected by ultrasound and 2 (1.8%) cases with minor ultrasound findings not confirmed at autopsy.^9^

Hence, when compared with above‐mentioned studies,^2^, ^5^, ^9^ our findings revealed lower rates of complete agreement between prenatal ultrasound and autopsy (40.2% vs. 72.6%, 74.1% and 87%, respectively) and higher rates of altered diagnosis after autopsy (9.8 vs. 1.6%) and disagreement between the methods (findings only on autopsy in 24.3% vs. 16.1% and 8.9% of cases, respectively). The present study was conducted at an earlier gestational week (<24 weeks of pregnancy) compared with other studies covering advanced gestational weeks, so the correlation between prenatal ultrasound and autopsy will be lower. In addition, anomalies such as lymphangiectasia, lack of uterus, anal atresia, ear anomaly, and oligodactyly can be more difficult to diagnose in early pregnancy by ultrasonography.

In a retrospective review of pregnancy terminations due to fetal anomaly performed between 1994 and 2009, the authors reported the full agreement between prenatal ultrasound and autopsy diagnoses in 91.7% of cases, along with a change in diagnosis (17.5%, particularly for musculoskeletal, neurologic, and multiple anomalies) and in recurrence risk (3.9%, particularly for neurologic, genitourinary, and multiple anomalies) based on autopsy findings.^4^

In fact, although several studies including fetuses from all gestational weeks, spontaneous abortions, and neonatal deaths emphasized a strong correlation between ultrasound and autopsy,^4^, ^5^ the studies specifically included fetal malformations detected at prenatal ultrasound in second‐trimester termination of pregnancy and revealed discrepancies between ultrasound and autopsy findings in about 40% of cases, emphasizing the importance of autopsy examination after every termination of pregnancy.^3^, ^10^, ^11^

In particularly, in a systematic review of 19 studies with 3534 fetuses on the correlation between fetal autopsy and ultrasound findings of fetal malformations that resulted in pregnancy terminations, the authors reported that autopsy findings confirmed prenatal ultrasound in 2401 (68.0%) fetuses, provided additional information in 794 (22.5%) fetuses, and did not confirm prenatal ultrasound in 329 (9.2%) fetuses.^12^ The highest agreement between autopsy and prenatal ultrasound was observed in central nervous system (79.4%) and genetic (79.2%) anomalies, followed by genitourinary (76.6%) and skeletal (76.6%) anomalies.^12^ The authors concluded that despite the high agreement between prenatal ultrasound and autopsy, a fetal autopsy is mandatory because it may provide additional findings or change the final diagnosis and genetic counseling in some cases.^12^

In the present study, autopsy revealed altered diagnosis of OICS syndrome (*n* = 1) and bladder exstrophy (*n* = 1) in two cases diagnosed with cloacal exstrophy on prenatal ultrasound, and OEIS syndrome (*n* = 1) and renal agenesis (*n* = 1) in two cases diagnosed with bladder exstrophy on prenatal ultrasound. Accordingly, given the remarkable difference in recurrence risk of bladder exstrophy (1/100 in the next offspring) versus OEIS syndrome, renal agenesis or cloacal exstrophy,^13^, ^14^ our findings emphasize the value of fetal autopsy in pregnancy terminations not only in terms of final diagnosis but also for the appropriateness of the recurrence risk assessment and the related genetic counseling for future pregnancies. Indeed, discrimination of cloacal or bladder exstrophy from OICS syndrome or OEIS syndrome is considered challenging in the prenatal period with accurate prenatal diagnosis of the syndromes being achieved in only half of cases.^15^, ^16^, ^17^, ^18^ Nonetheless, although it appears to be a milder anomaly than OICS syndrome, cloacal exstrophy per se has similarly high morbidity and mortality.^15^ Accordingly, in our case series, the altered diagnosis after autopsy was mostly related to identification of additional anomalies that reveal a diagnosis of a syndrome complex (i.e., OEIS or OICS syndrome), indicating that autopsy findings were at least as severe as the sonographic findings and the management was correct.^1^ Similarly, in a retrospective study on assessment of prenatal ultrasound and autopsy findings in nine fetuses with OEIS complex, the main findings on prenatal ultrasound were reported to be omphalocele, skin‐covered lumbosacral neural tube defect, non‐visualized bladder, and limb defects, whereas autopsy added findings that prenatal ultrasound failed to detect were the abnormal genitalia, bladder exstrophy, and anal atresia.^19^

Indeed, a diagnosis made in a tertiary center is considered to reveal a higher detection rate of fetal malformations, mainly concerning functional defects, and a smaller number of false negatives,^3^ whereas the inconsistencies between prenatal ultrasound and autopsy findings are considered likely to be related to a higher likelihood of perinatologists to diagnose a major anomaly rather than minor anomalies, which are in fact considered very important given that they may alter the diagnosis as a syndrome versus an isolated malformation.^2^, ^7^, ^10^, ^20^ Notably, prenatal ultrasound revealed isolated urogenital anomaly in 30.5% cases in our study, whereas much higher rates for isolated anomaly (76%) were reported in a study indicating a higher agreement rate between prenatal ultrasound and autopsy in the diagnosis of urinary system anomalies than identified in our study.^9^

In the present study, megacystis (*n* = 14 and 7, respectively), bilateral renal agenesis (*n* = 9 and 5, respectively), and infantile polycystic kidney (*n* = 8 and 5, respectively) were the most common urogenital anomalies detected on prenatal ultrasound overall and also were the three most common diagnoses confirmed by both prenatal ultrasound and fetal autopsy. These findings seem consistent with consideration of either renal agenesis or various forms of cystic renal disease to be the most common urogenital anomalies resulting in pregnancy terminations and they were either suspected or correctly diagnosed prenatally.^9^

In our series, considering the disagreement between ultrasound and autopsy, renal agenesis (*n* = 5), horseshoe kidney (*n* = 4), and multicystic dysplastic kidney (*n* = 4) were the most common ones among the 20 anomalies detected only on autopsy, while horseshoe kidney (*n* = 3) and megacystis (*n* = 2) were the most common ones among the nine anomalies detected only on ultrasound. These findings support the consideration of isolated unilateral kidney lesions (i.e., agenesis, hypoplasia, or dysplasia) to escape detection more often than bilateral lesions, given that they do not cause amniotic fluid alterations that trigger the awareness for a renal anomaly.^9^

In particular, in our series, horseshoe kidney on autopsy was falsely interpreted as renal agenesis on ultrasound in one case, which supports the consideration of horseshoe kidneys to be difficult to detect on ultrasound because the connection may be missed in the two‐dimensional plane.^12^, ^21^ Also in our series, polycystic kidney disease on autopsy was falsely interpreted as multicystic disease on ultrasound in one case, which seems notable given that distinguishing between polycystic disease of the kidneys and multicystic renal dysplasia is in fact considered to be difficult on gross examination at autopsy.^9^ Notably, to distinguish between autosomal recessive polycystic kidney disease and multicystic dysplastic kidney is considered important, because recurrence risk is 3% in cystic renal dysplasia in contrast to 25% in autosomal recessive polycystic kidney disease^22^; multicystic dysplastic kidney is also suggested to deserve special attention because of the increased risk of additional renal or extra‐renal anomalies in this condition.^23^

Complete agreement between fetal ultrasound and autopsy diagnoses was evident in only 40.2% of our cases. Partial agreement with additional minor findings on autopsy in 14.6% of cases seems to be explained by the concomitant presence of oligohydramnios in urogenital anomalies, which may cause suboptimal assessment of ultrasound imaging due to reduced image quality. Additional minor findings may also be overlooked because of maternal abdominal fat tissue as well as the primary focus of prenatal assessment on the major anomaly.

In eight pregnancy terminations, the final diagnosis as well as the risk of recurrence have changed after autopsy examination. This seems to emphasize the importance of avoiding dilemmas in pregnancy terminations by informing parents before termination about the potential discrepancy between fetal ultrasound and autopsy findings and the likelihood of ultrasound‐based anomaly diagnosis not to be confirmed by autopsy in some cases. Nonetheless, it should also be emphasized that autopsy is the only method to test the accuracy of prenatal ultrasound‐based diagnosis and hence is the reference standard to provide genetic counseling regarding the likelihood of recurrence in subsequent pregnancies in the case of a confirmed diagnosis of fetal anomaly.

The major strength of the present study seems to be the inclusion of pregnancy terminations before the 24th week of pregnancy for congenital anomalies, specifically urogenital anomalies, and the consideration of our center as a reference tertiary care center with fetal ultrasound and fetal autopsy examinations performed by the same perinatologist and perinatal pathologist for at least 16 years. Certain limitations to this study should be considered. However, our findings support that fetal autopsy (providing information about abnormalities of clinical importance, genetic guidance, and epidemiologic data) is of vital importance in improving the quality of prenatal ultrasound (dynamic examination providing additional information about functional anomalies) and complementing the prenatal diagnosis.^1^, ^2^, ^3^, ^5^, ^9^

In conclusion, our findings revealed complete to partial agreement between prenatal ultrasound and autopsy diagnoses on fetal urogenital anomalies in half of pregnancy terminations, whereas autopsy findings that revealed either altered diagnosis or additional minor anomalies were at least as severe as the sonographic findings, emphasizing that the information and advice given to the parents and the management was correct. Accordingly, our findings indicate fetal autopsy to be a method of crucial importance in complementing prenatal diagnosis, which may add valuable information that may improve subsequent pregnancy management and counseling of parents, and hence prenatal ultrasound and fetal autopsy should be regarded as complementary techniques.

## CONFLICT OF INTEREST

The authors have no conflicts of interest relevant to this article.

## AUTHOR CONTRIBUTIONS

Ozge Ozdemir, Figen Aksoy, and Cihat Sen contributed to conception/design of the research; acquisition, analysis, and interpretation of the data; and drafted the manuscript; Ozge Ozdemir and Cihat Sen critically revised the manuscript. All authors read and approved the final manuscript.
